# The Colorful Sex Chromosomes of Teleost Fish

**DOI:** 10.3390/genes9050233

**Published:** 2018-05-03

**Authors:** Verena A. Kottler, Manfred Schartl

**Affiliations:** 1 Department of Physiological Chemistry, Biocenter, University of Wuerzburg, 97074 Wuerzburg, Germany; verena.kottler@uni-wuerzburg.de; 2 Comprehensive Cancer Center Mainfranken, University Clinic Wuerzburg, 97080 Wuerzburg, Germany; 3 Hagler Institute for Advanced Study and Department of Biology, Texas A & M University, College Station, TX 77843, USA

**Keywords:** teleost fish, sex chromosomes, coloration, pigment pattern, sexual conflict, sexually antagonistic genes

## Abstract

Teleost fish provide some of the most intriguing examples of sexually dimorphic coloration, which is often advantageous for only one of the sexes. Mapping studies demonstrated that the genetic loci underlying such color patterns are frequently in tight linkage to the sex-determining locus of a species, ensuring sex-specific expression of the corresponding trait. Several genes affecting color synthesis and pigment cell development have been previously described, but the color loci on the sex chromosomes have mostly remained elusive as yet. Here, we summarize the current knowledge about the genetics of such color loci in teleosts, mainly from studies on poeciliids and cichlids. Further studies on these color loci will certainly provide important insights into the evolution of sex chromosomes.

## 1. Introduction

### 1.1. Sex Determination, Sex Chromosomes, and Sexually Antagonistic Genes

Teleost fish display a spectacular diversity of color patterns, making them one of the most colorful vertebrate groups. The pigment patterns of teleosts range from inconspicuous camouflage to vividly colored ornaments, which have puzzled scientists for almost a century [1,2,3,4,5,6]. While the coloration of females and males of well-studied species such as the zebrafish (*Danio rerio*) and the medaka (*Oryzias latipes*) is mostly similar, the sexes of many other teleost fish can easily be distinguished from each other by their pigmentation. This sexual dimorphism can be either permanent or transient; in the latter case, it is, for instance, restricted to intra- or interspecific competition, to courtship, or to other periods during reproduction [7,8,9,10,11]. Color differences between sexes are usually under strong selection as they play a major role in the adaptation and evolution of a species [10,12,13,14,15].

When selection favors divergent phenotypes in females and males, a substantial sexual conflict can arise if the trait providing optimal fitness for females decreases male fitness and vice versa, hence is subjected to sexually antagonistic selection [16,17,18]. Usually, such sexual conflicts are solved by the sex-limited or -biased expression of the underlying loci, but sexual conflict may also contribute to speciation [10,17,19,20,21,22]. To achieve sex-limited or -biased expression patterns, one possibility is that these loci become located in a region of the genome that is unique to the sex for which they are advantageous. Such loci are therefore frequently found in the sex-specific regions of heterogametic sex chromosomes, where recombination is very low or absent. Within the non-recombining region of the sex chromosomes, sexually antagonistic loci can be inseparably linked to the sex-determining locus, together forming a “supergene”, which solves the sexual conflict by ensuring that such loci occur in only one of the sexes [23,24]. Another possibility is that the expression of sexually antagonistic loci comes under the regulation of sex-specific factors, for instance, hormones, that are under the control of the primary sex-determining locus on the sex chromosome [25,26,27,28].

Sexually antagonistic genes or alleles are believed to be of outstanding importance for sex chromosome evolution [29,30,31,32]. The current state of knowledge predicts that sexually antagonistic loci can be maintained if they arise in the region of suppressed recombination surrounding the sex-determining locus [29,30]. On the other hand, they are predicted to even constitute major drivers of sex chromosome evolution by promoting the establishment of suppressed recombination around a novel, adjacent sex-determining locus [31]. Sexually antagonistic loci might also stabilize the transposition of an ancestral sex-determining gene to an autosome and might help to maintain multiple sex-determining factors in species that lack heteromorphic sex chromosomes [31]. When novel male- or female-beneficial loci emerge at the borders of the sex-determining region where substantial cross-over still occurs, selection favors the expansion of the non-recombining region along the sex chromosome [29]. This positive feedback loop leads to the formation of distinct evolutionary strata along the sex chromosomes that differ in their genetic divergence [29,33,34].

As coloration is an easily observable trait, it is not surprising that already in 1907, it was proposed that the lacticolor pigment variant of the magpie moth (*Abraxas grossulariata*) is directed by a genetic locus on the sex chromosomes [35]. In fish, Aida first described a red color locus named R residing on the X- and Y-chromosome of medaka in 1921 [1]. This was followed by the publication of a collection of classical papers on sex-linked pigmentation in fish. The loci directing sex dimorphic coloration in teleosts provide prime examples of sexually antagonistic genes, as Fisher and Winge already noted when examining guppy (*Poecilia reticulata, Poecilia wingei, Poecilia obscura*) pigmentation in the 1920s and 1930s [5,6,32].

Teleosts show great variation in the way that the female and male sex is determined, which, depending on the species, can be triggered by environmental or genetic mechanisms [36,37,38,39,40,41,42,43]. Even closely related species can have different sex determination mechanisms [37]. As sexually antagonistic genes can be linked to Y- and W-chromosomes or be present in polyfactorial systems, the study of such genes is particularly illuminating in this group.

### 1.2. Pigmentation in Fish

The color pattern of vertebrates is generated by pigment cells (chromatophores), which are derived from the neural crest [44,45]. At least five different pigment cell types have been described in fish: black melanocytes, also called melanophores (for definition see [46]), orange xanthophores, white leucophores, iridescent iridophores, and blue cyanophores [46,47,48,49]. In contrast, mammals have lost all chromatophore types except melanocytes [46]. A plethora of pigmentation genes have been described in mice (*Mus musculus*; for an overview see http://www.espcr.org/micemut/), but taking into account the teleost-specific whole genome duplication, the persistence of a much wider array of pigment cell types, and the spectacular pigment patterns, the genomes of teleosts are predicted to harbor many more color loci than the ones of mammals [50,51]. Yet, most pigmentation studies have been conducted in mice and humans (*Homo sapiens*) and only a few pigmentation genes have been characterized in fish [52,53,54,55,56].

When describing color loci, it is important to group them according to their function: (i) pigment synthesis; (ii) pigment cell development; and (iii) pigment pattern loci. Examples of pigment synthesis genes are *tyrosinase (tyra* and *tyrb*) and *6-pyruvoyltetrahydropterin synthase* (*pts*) that are required for the synthesis of eumelanin and orange pigments within the melanocytes and xanthophores, respectively [51,57,58,59]. Genetic loci that direct pigment cell development affect, for example, chromatophore differentiation and size. Such genes are, for instance, *colony-stimulating factor 1 receptor a* (*csf1ra*), which is required for xanthophore development, and *kita*, which directs the development of certain melanocyte populations [60,61,62,63,64]. While the biochemical pathways of eumelanin synthesis and some aspects of chromatophore development are well studied, only few pigment pattern genes have been identified at the molecular level so far and hardly any that underlie sexually dimorphic pigmentation. Pigment patterns are generated by the spatial and temporal distribution of the chromatophores, which can be mediated by interactions between the pigment cells [52,65,66,67]. For instance, zebrafish *connexin41.8* mutants form spots instead of stripes as the interaction between melanocytes and xanthophores is impeded by a gap junction defect [67,68]. It has to be mentioned that many of the so far discovered color genes perform multiple, overlapping functions. Zebrafish and guppies lacking xanthophores due to a mutation in *csf1ra*, for instance, display a severely altered pigment pattern as they lack orange pigments and the cues from the xanthophores that the melanocytes need to position themselves correctly in the skin [61,63,64].

Color loci are widespread in the genomes of all vertebrates and statistically, several such loci are expected to be present on each chromosome. For instance, *solute carrier family 45 member 2* (*slc45a2*) and *sepiapterin reductase a* (*spra*) are present on the sex linkage group of the guppy, which are required for pigment synthesis in male and female chromatophores [51,69,70]. Thus, sex chromosomes will always carry some color genes, some of which might be later recruited for a sex-specific function. Alternatively, color loci might have been translocated to the proto-sex chromosome or even to a sex chromosome in a more advanced evolutionary stage. Synteny analysis of color genes between species that differ in their sex-linkage groups should clarify this issue, but such studies have not been conducted thus far.

Color loci that are sex chromosome-linked can be located in either the pseudoautosomal region or the sex-determining region of the sex chromosomes (for instance, [71]). The ones in the pseudoautosomal region can still undergo cross-over, although recombination rates might be reduced depending on the genomic structure and distance to the sex-specific region, while the ones close to the sex-determining locus occur in the non-recombining stratum that is specific for one of the sex chromosomes, for instance, the Y-chromosome (“Y-specific genes”) [71,72,73]. Here, we will review the current knowledge about color loci located on the sex chromosomes that underlie the sexually dimorphic pigmentation of teleosts, a topic that is intrinsically tied to sex chromosome evolution. We will especially focus on the family of live-bearing fish, the Poeciliidae, as many studies have addressed their sex chromosome and color pattern diversity.

## 2. Guppy

Some of the most famous and best studied examples of Y-specific color loci are the pigmentation genes underlying the vivid orange, black, green, and iridescent spots and stripes of male guppies. Guppies are small live-bearing teleosts, whose coloration has been investigated since the 1920s [5,6,74]. While guppy females are inconspicuously colored, male coloration in all three species of guppies (*P. reticulata*, *P. wingei*, and *P. obscura*) is highly polymorphic, to the extent that each male can be identified by its individual color pattern [12,75]. A wealth of studies on guppy ecology and behavior has demonstrated that male coloration as well as life-history traits like body shape and brood size covary with predation intensity [13,76,77,78]. When major predators are present, dull male coloration protects against predation, but in low predation environments, sexual selection in the form of female choice leads to an increase in male colorfulness within a few generations [12,79,80]. This indicates that sexual selection operates under the constraints of natural selection, which can limit the extent of pigmentation when exaggeration of this trait becomes a threat. Rare male color morphs seem to be maintained within guppy populations by negative frequency-dependent selection [80]. Female guppies are especially attracted to males displaying a pronounced orange coloration, which might be derived from a sex-independent sensory bias for orange [81].

**Figure 1:**
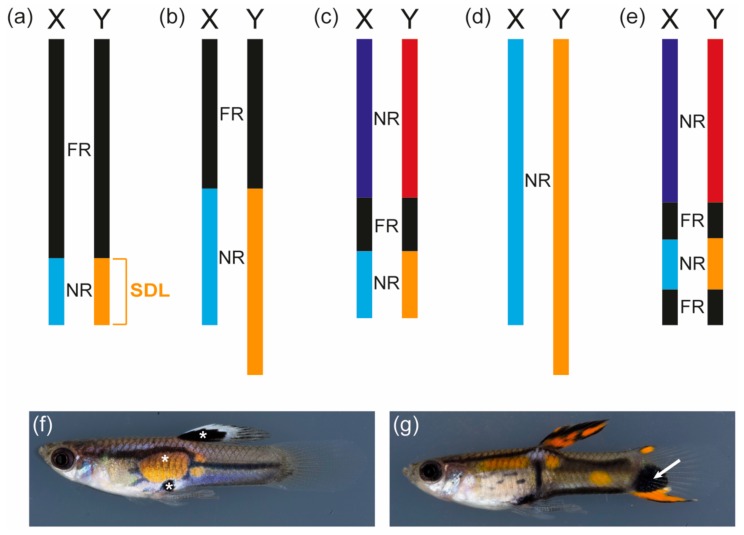
Guppy sex chromosomes. (**a**–**e**) Schematic representations of guppy sex chromosomes proposed by different studies (blocks only roughly drawn to size). (**a**) Inferred from cross-over frequencies of male pigmentation traits; summarized in 1947 [71]; (**b**) Deduced from cytological evidence in 2001 [85]; (**c**) Derived from the analysis of genetic markers in 2009; no marker in the light blue/yellow NR could be found, which is why the size of this region is unknown [82,83]; (**d**) Based on cytological evidence in 2014; no clear chiasma structure between X and Y could be detected [75]; (**e**) Proposed based on a cytological study in 2015; an ornamental guppy strain was investigated [84]. The distal yellow block contains the male sex-determining locus (SDL) and Y-specific color loci, for instance, maculatus (**f**), pauper, and armatus. The structure of the sex chromosomes varies greatly between guppy populations. (**f**) White asterisks mark the Y-specific maculatus traits described in the text. Total length of the male: 2.4 cm; (**g**) Male with an EnCCFR (Cumaná) Y-chromosome featuring a black spot (white arrow) in the tailfin, which is associated with a constriction of the X-chromosome. Total length of the male: 2.5 cm. Guppy pictures courtesy of Christine Dreyer. FR (black): freely recombining region; NR (light blue, yellow, dark blue, red): non-recombining region or region with reduced recombination.

Several cytological and molecular studies have investigated the sex chromosomes of the guppy, providing extensive information on the genomic organization of the sex linkage group (Figure 1a–e). The guppy is the only teleost fish in which such a plethora of studies on the sex chromosomes have been conducted. The sex-determining locus of the guppy has been genetically mapped to a male-specific non-recombining region at the distal end of the Y-chromosome, which is predicted to be at least 0.5 to 5 million years old (Figure 1a–d) [75,82,83]. Based on genetic markers, a pseudoautosomal region with reduced recombination at the proximal end of the chromosome is separated from the distal region of the Y by a freely recombining part (Figure 1c) [82]. Based on an immunocytological analysis, a recent study postulated that the most distal tip of the Y-chromosome contains a second freely recombining area, but this was based on the analysis of an ornamental guppy strain (Figure 1e) [84]. Several studies have demonstrated that the Y-chromosomes of different guppy strains vary in their heterochromatin content, which had already been predicted by Winge in 1922 [75,82,85,86]. This can even be observed in chromosome spreads where the Y-chromosomes of different guppy strains differ considerably in their length [75]. YY males of the guppy are viable as long as the Y-chromosomes are derived from different strains, which provides further evidence that individual Y-chromosomes differ considerably and might have lost different genetic elements or acquired lethal factors [24,75,87]. So far, no genetic marker specific for the Y-chromosomes common to all guppy strains has been identified [75,83].

In 1922 and 1927, Winge described 18 color loci found in guppies, of which nine were Y-specific and all but one showed sex chromosome-linked inheritance. All of the loci characterized by Winge can be classified as sexually antagonistic pigment pattern genes as they direct the development of colorful spots and stripes that only occur in guppy males. One example of a strictly Y-specific locus is maculatus, which controls the formation of a black spot in the dorsal fin and an orange and a black spot at the center of the body (Figure 1f) [5]. During 60 years of breeding, cross-over of maculatus was only observed once, suggesting that this locus forms a supergene with the sex-determining locus on the Y-chromosome or even directs both male sex determination and ornamentation [5,24,71]. It remains to be determined whether other Y-specific color loci, for example, armatus and pauper, represent alleles of the same locus as maculatus and whether these loci are indeed located at the distal tip of the chromosome or are scattered within the heterochromatic block observed in this region [6,71,75]. Surprisingly, guppy XY females of the maculatus strain show the same, though fainter, black spot in the dorsal fin as maculatus males, but none of the other pattern elements ([87,88] and personal observation). This suggests that the black spot in the dorsal fin, as expected from a Y-specific trait, is not under hormonal control, while the other pattern elements directed by maculatus are still dependent on male hormonal cues. This might indicate that maculatus was once located in the pseudoautosomal region of the sex chromosomes or on an autosome, where many ornaments of guppy males are encoded [75,83,89,90].

An example of such a male color pattern is the black spot occurring in the tailfin of some guppy populations (Figure 1g) [75,83,90]. This trait is directed by a locus in the pseudoautosomal region and is associated with an X-chromosome constriction [75,90]. The highest recombination frequencies found between a marker on the sex chromosomes and the sex locus was 10% in the pioneering work by Winge and his colleagues and 2.3% in a more recent quantitative trait locus (QTL) study, suggesting that cross-over between the guppy X and Y is low but substantial [71,82]. QTL analyses furthermore suggest that coregulation by autosomal and pseudoautosomal loci considerably affects male color pattern development [83,90].

Depending on the population, guppy females develop some spots and stripes when treated with testosterone [26,27,28]. This reveals that testosterone, whose high level is a consequence of male development triggered by the sex-determining locus, causes the male-limited expression of hormone-sensitive pigment pattern loci situated outside of the Y-specific region. Guppies are therefore a fascinating example of a species where a sexual conflict is solved by both supergene formation and sex-specific hormones. Intriguingly, the extent of Y-specific male coloration decreases within a few generations when guppy males are transferred from high- to low-predation environments and males from low-predation environments in general show a higher amount of (pseudo-) autosomal-linked coloration [26,27,28]. This suggests that the sex chromosomes of guppies can adapt rapidly to changing selective pressures, most likely by the expansion of the non-recombining region or by translocation of the color loci between the male-specific and the pseudoautosomal region [26,27,91].

Although genomic resources have recently become available for the guppy, only the autosomal color loci responsible for the non-sex-specific golden, blue, and blond coloration have been already identified [61,69,91,92,93]. The underlying genes, *kita*, *csf1ra*, and *adenylate cyclase 5* (*adcy5*) affect melanocyte and xanthophore development and are most likely indirectly required for male pattern formation [61,92]. The composition of the male-limited and Y-specific pigment pattern loci as iconic as maculatus, however, remains elusive.

## 3. Other *Poecilia* Species

Five distinct male color morphs of *Poecilia parae* have been described, which also differ in body size and mating behavior [94,95]. In the “red”, “yellow”, and “blue” morph, the pigment cells are arranged in horizontal stripes on the body, while the “parae” morph displays a colorful stripe on the tailfin and black vertical bars on the caudal peduncle [95]. Additionally, some males look like females and do not show any male pattern elements (“immaculata” morph) [95]. Females seem to prefer the yellow and red color variants, suggesting that this polymorphism is shaped by sexual selection [95,96]. Crosses demonstrated that the male pattern is Y-specific, which indicates that it is tightly linked to the sex-determining locus and that the different color morphs might be generated by allelic variation [95]. Conversely, male red coloration in the sister species of *P. parae*, *Poecilia picta*, is not a Y-specific trait and female *P. picta* do not prefer red males [97,98]. This demonstrates that the forces shaping coloration can vary greatly even between closely related species.

## 4. Swordtails and Platyfish

**Figure 2:**
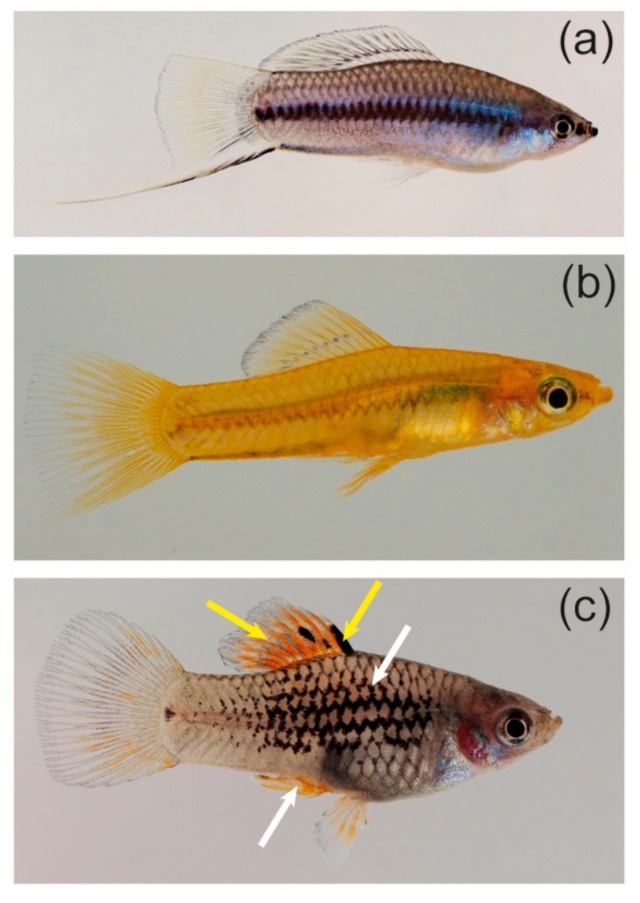
Color patterns of *Xiphophorus* fish. (**a**) Blue male with a yellow tailfin of *Xiphophorus nigrensis*. Total length of the fish: 6 cm; (**b**) Yellow male of *Xiphophorus pygmaeus*. Total length of the fish: 3 cm; (**c**) *Xiphophorus maculatus* male with two different macromelanocyte and red-yellow (RY) patterns. White arrows: Y-chromosomal macromelanocyte stripes with linked RY pattern in the anal fin. Yellow arrows: X-chromosomal macromelanocytes and linked RY pattern in the dorsal fin. Total length of the fish: 3 cm.

The sexually dimorphic pigmentation of the swordtail species *Xiphophorus multilineatus*, *Xiphophorus nigrensis*, and *Xiphophorus pygmaeus*, which have an XX/XY sex determination system, has been intensively studied. Males of all three species show either a blue or a yellow body coloration, which is due to allelic variation at a Y-chromosomal locus (Figure 2a,b) [99,100]. Males carrying the *+* allele are blueish, males with the *cp* allele show a blue body pigmentation with a yellow tailfin, and fish with the *con* allele are completely yellow (Figure 2a,b) [99,100]. Yellow *X. pygmaeus* males (Figure 2b) perform greater chasing behavior towards females and are more aggressive, but females prefer blue males [101]. Most likely, this explains why the frequency of yellow *X. pygmaeus* is stabilized at around 13–25% in natural populations [100,101]. Fascinatingly, the color locus forms a supergene with the so-called pituitary locus (P locus), which controls male size and behavior, on the Y-chromosomes of these swordtails [101,102,103]. The pronounced male size and behavior polymorphisms are caused by *melanocortin receptor 4* (*mc4r*) allelic and copy number variation at the P locus [103]. For instance, *con* only occurs in combination with the *s* allele of the P locus, which leads to early maturity and small body size in male *X. nigrensis* and *X. multilineatus* [102]. So far, it remains unclear whether this supergene resides in the non-recombining male-specific region of the Y or is closely linked to it in a region of reduced recombination. In *X. nigrensis* from Rio Coy (San Luis Potosi, Mexico), the Y-chromosome with the *s* allele is also supposed to contain a genetic locus suppressing the formation of male-specific vertical bars on the body, which might be associated with male mating success [104,105]. In another species, *Xiphophorus cortezi*, these vertical bars occur in both sexes, which demonstrates that similar patterns can be subjected to different regulatory mechanisms in closely related species [106].

Many *Xiphophorus* species are polymorphic with black pigment patterns that are composed of a peculiar type of melanocytes, the macromelanocytes (previously called macromelanophores; Figure 2c). Macromelanocytes usually reach a diameter of 300 to 500 µm and are therefore much larger than the regular melanocytes, which make up the grayish background coloration of the fish and have a size of around 100 µm [107]. The diverse macromelanocyte patterns, which can manifest as pepper-and-salt-like spotting on the body, blotches on the flanks, or stripes and spots in the dorsal and caudal fins, are encoded by an allelic series of the macromelanocyte-determining locus (Mdl) [107,108,109,110]. Mdl has been shown to be located on the sex chromosomes of several species including the well-studied Southern platyfish (*Xiphophorus maculatus*), in which sex is determined by three different homomorphic chromosomes (W, Y, X) [85,107,111,112]. In *X. maculatus,* Mdl alleles are encoded on the X- and Y-, but not on the W-chromosome [110]. On the *X. maculatus* X and Y, Mdl is intimately linked to the *xmrk* oncogene, which is responsible for melanoma formation from the macromelanocyte spots in interspecific hybrids [107,113,114]. In other *Xiphophorus* species, however, Mdl alleles do not necessarily contain *xmrk* [107]. The *xmrk* oncogene is a mutated, constitutively active version of the *epidermal growth factor b* (*egfrb*) gene, which is a necessary component of Mdl in platyfish as loss-of-function mutation of *xmrk* leads to the loss of the macromelanocyte pattern [107,113,115]. Sex chromosomal cross-overs that involve Mdl occur, but at a very low rate (far below 0.001), indicative of a close linkage to the sex-determining region [110,116]. Intragenic cross-overs can even occur within *xmrk* [116]. The fact that the *xmrk*/Mdl locus is enriched with transposable elements is in line with its location in the vicinity of the sex-determining gene [117].

Some of the Mdl patterns occur together with red, orange, or yellow pigmentation of the iris, fins, or regions of the trunk (Figure 2c) [110,116]. It is unclear whether these color patterns are the result of the expression of another separate, closely linked locus (named RY for red-yellow in the literature) or are encoded by specific Mdl alleles [110,116].

In contrast to the sex-chromosomal pigment patterns of the guppy and *P. parae,* the patterns of platyfishes are also expressed in females, although they are generally much more intense in mature males (reviewed in [118]). In *X. cortezi,* the macromelanocyte pattern Sc (spotted caudal) is clearly under sexual selection as females prefer males with larger spots [119]. The situation in *Xiphophorus* may represent a situation where a sex chromosome-linked pigmentation pattern only has a mildly beneficial effect for one sex and a similar mildly antagonistic effect on the other sex, or where the sexual conflict has not been solved effectively.

## 5. Eastern Mosquitofish

Female and male eastern mosquitofish (*Gambusia holbrooki*) are usually well camouflaged by their grayish coloration. However, in some populations, up to 1% of eastern mosquitofish males display a conspicuous melanic color pattern, which is generated by large macromelanocytes [120,121,122,123,124,125]. The expression of this color pattern is constitutive, but a temperature-dependent allele has also been described as low temperatures trigger the appearance of the macromelanocytes in some *G. holbrooki* populations [121,122,124,126]. Male melanic coloration is linked to a number of other traits including increased aggression towards competitors and females, and a larger body and intromittent organ size [122,126,127]. Mark-recapture and mesocosm studies have demonstrated that the melanic phenotype provides a survival advantage when predators are present as long as the phenotype is rare [122,123,125]. Female eastern mosquitofish prefer melanic males if they are derived from populations where such males occur [128].

In contrast to its sister species *Gambusia affinis*, no heteromorphic sex chromosomes are discernible in *G. holbrooki* male and female chromosome spreads [129]. As the melanic color pattern is transmitted from father to son, the melanic locus is presumed to be Y-specific, suggesting that the eastern mosquitofish has an XX/XY sex determination system [121,124]. This demonstrates that sex chromosome-specific color loci can occur on seemingly homomorphic sex chromosomes. Melanic *G. holbrooki* females have very rarely been observed in the laboratory and could be explained by recombination between the X- and the Y-chromosome or an incidence of XY male to female sex reversal [121,130]. Whether the behavior and life-history traits associated with the melanic phenotype are mediated by the locus itself or by loci nearby cannot be evaluated yet and a genetic pigment cell development locus directing the formation of macromelanophores in other species than *Xiphophorus* remains to be found.

## 6. Cichlids

Cichlids are famous for their highly variable and sexually dimorphic coloration. Mostly driven by sexual selection, the color patterns of cichlids evolve rapidly and contribute greatly to the diversification and speciation of this species-rich group [9,15,20,131,132]. Sex determination in cichlids is multifactorial and not well understood. For instance, sex in certain Lake Malawi (Africa) cichlids seems to be determined by epistatic loci on four different chromosomes and in the cichlid *Lithochromis rubripinnis* from Lake Victoria (Africa), female-specific B-chromosomes occur [38,133].

One example for a sexually antagonistic color locus is thought to be provided by the orange-blotch (OB) pattern, which frequently occurs in females belonging to the group of haplochromine cichlids in Lake Malawi and Victoria [10,21,134]. OB females display black spots at various positions on the body, which could provide camouflage in their rocky habitats [10]. The OB phenotype is very rare in cichlid males where it might interrupt the nuptial coloration and hence might be disadvantageous [10]. In some *Metriaclima* and *Labeotropheus* species from Lake Malawi, OB is linked to a dominant female-determining locus on chromosome 5, while in other species, sex is determined by a locus on chromosome 7, mediating a XX/XY system [134]. In *Metriaclima pyrsonotus*, both systems occur [134]. This may constitute an intriguing example of the evolution of a W sex chromosome that has replaced, or is about to replace an existing XX/XY system [10,21,133,134,135]. Fine mapping of the OB locus linked the mottled appearance to the transcription factor Pax7, whose overexpression leads to the development of fewer but larger melanocytes [10]. In Lake Victoria cichlids, OB is most likely caused by a different locus of unknown identity [10,136]. In Lake Tanganyika (Africa), males of several cichlid species display a yellow or blue fin polymorphism, which is presumably caused by a color locus in a region with reduced recombination [137].

In addition to the sex-specific color loci, the involvement of several, presumably autosomal, loci has been implicated in the development of sexually dimorphic coloration in cichlids. One example of this is *csf1ra*, which is expressed in the egg spots of haplochromine cichlids and seems to be under positive selection in this lineage [138]. Moreover, *four and a half LIM domain protein 2b* (*fhl2*) is expressed in the iridophores within the egg spots, which is facilitated by a regulatory change unique to haplochromines [139]. In *Pseudotropheus saulosi*, *coatomer protein complex subunit zeta-1* (*copz-1*) might be involved in the development of color dimorphism [140]. How these loci contribute to male pattern elements as specific as egg spots still needs to be determined.

## 7. Medaka

Medaka has an XX/XY sex determination system where the male sex is determined by a Y-specific duplicate (*dmrt1bY*) of the transcription factor Doublesex and mab-3 related transcription factor 1b (*dmrt1a*) [141,142,143]. The Y-chromosome is around 5 to 10 million years old [144]. Despite small phenotypic differences, medaka males and females look very similar. Two recessive sex-linked color phenotypes have been described in this species so far, which have not been observed in wild populations and were most likely maintained by hobby breeders. White (r, colorless xanthophore) is located in the pseudoautosomal region of the sex chromosomes, as is leucophore free (lf), whose recombination frequency with the sex-determining locus averages 2.2% [1,143,145,146]. In the Hd-rR strain of medaka, females are white (X^r^X^r^) and male fish are red (X^r^Y^R^), while in the Qurt strain, females lack leucophores, as they are homozygous for lf [143,145,146]. White and leucophore free have yet to be cloned and presumably constitute examples for color loci that were “captured” coincidentally by a nascent sex-determining locus nearby.

## 8. Conclusions

Sex chromosomes are a fascinating part of the genome, posing intriguing questions at the interface of evolution and genetics. Establishment of reduced recombination constitutes one of the milestones of sex chromosome evolution, making these regions hotspots for the emergence of new alleles and the accumulation of repetitive and transposable elements. Sexually antagonistic color loci in close linkage to the sex-determining locus can contribute greatly to the development and divergence of sex chromosomes but are notoriously difficult to study at the molecular level due to the complex and usually repeat-rich genomic structure of the region in which they are located. Although one would predict that sexually antagonistic pigmentation genes should eventually become tightly linked to the sex-determining locus in the non-recombining region, such a situation has so far only been observed in a few teleost species. A simple reason for this might be that the sex chromosome systems of teleosts are considered to be very young [147]. Emerging model systems for coloration, for instance, killifish, will certainly soon provide more examples of such sex chromosome-linked loci [148].

New genome sequencing and mapping techniques have recently led to the discovery of the autosomal pigmentation supergenes that most likely underlie the mimicry in *Papilio* butterflies and the male mating phenotypes of the ruff (*Philomachus pugnax*) [149,150,151]. They have also provided great insights into the structure of autosomal color supergenes in *Heliconius* butterflies, emphasizing the importance of advanced sequencing technology to identify breakpoints and chromosomal rearrangements in the genome [152,153]. Pinpointing the genetic loci responsible for the diverse sexually dimorphic coloration of teleost fish is challenging as we have only scarce knowledge about the nature of such loci. While it might be possible that some encode transcription factors like Pax7, many others probably do not belong to the group of “classical”, well-studied color genes and might even be non-coding RNAs. Especially in diverged sex chromosomes with large regions of repressed recombination, “novel” color loci, for which a function in pigmentation has not been described yet, will be difficult to recognize. Discovering such loci is worth the effort though as they will provide spectacular—and colorful—insights into some of the most fascinating biological processes.
